# Supplementary material for: Overexpression of the RNA-binding protein NrdA affects global gene expression and secondary metabolism in Aspergillus species
Source: mSphere. 2025 Jan 24;10(2):e00849-24. doi: 10.1128/msphere.00849-24 (PMC11852746; doi:10.1128/msphere.00849-24)
Supplement: Supplemental Figures and Tables — Fig. S1 to S8; Tables S1 and S2. [file msphere.00849-24-s0002.pdf]

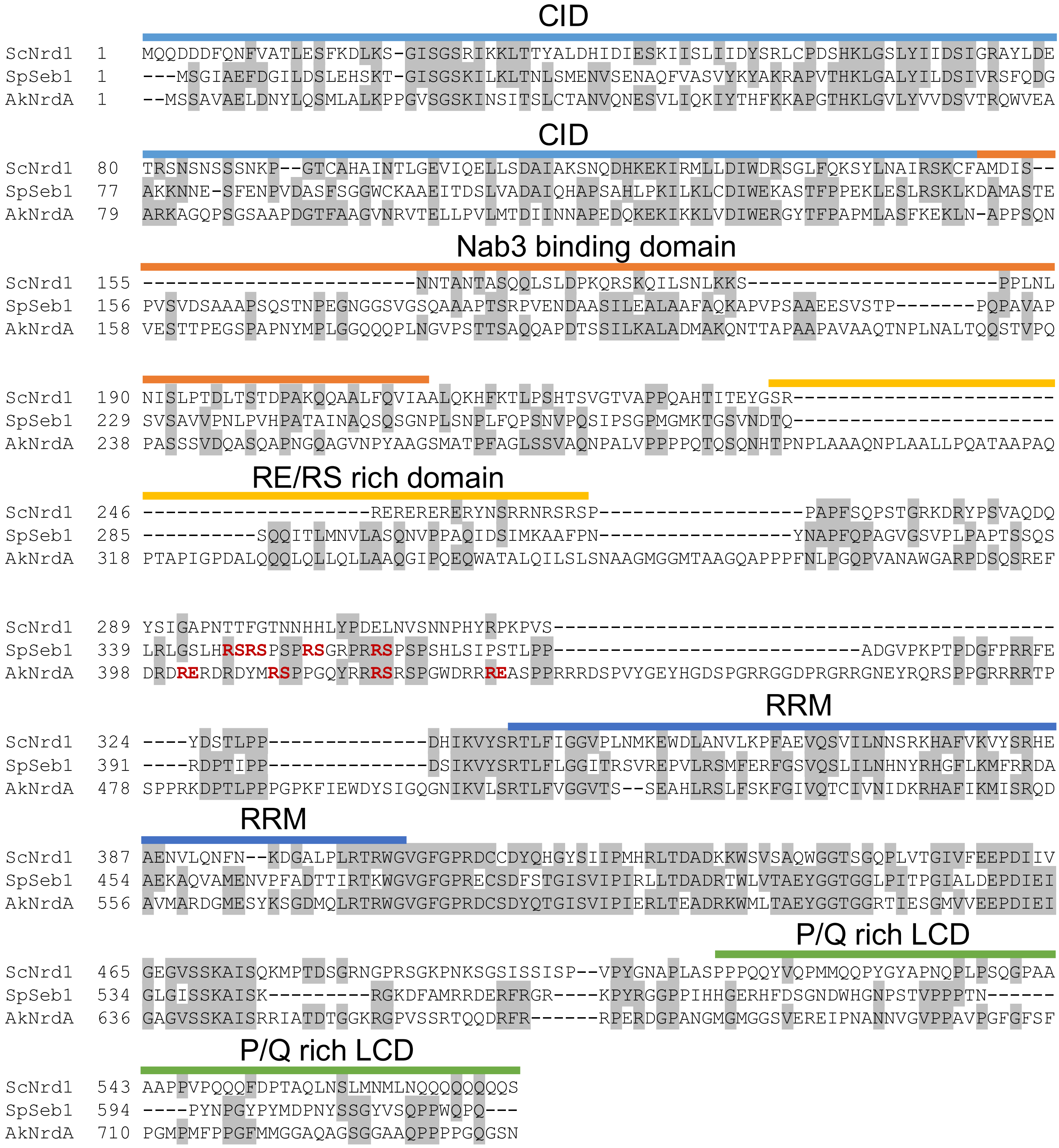

Supplementary Figure S1. Aligned amino acid sequences of *Saccharomyces cerevisiae* Nrd1 (ScNrd1), *Schizosaccharomyces pombe* Seb1 (SpSeb1), and *Aspergillus luchuensis* mut. *kawachii* NrdA (AkNrdA). Putative intron sequences of *A. kawachii nrdA* were confirmed by transcriptome sequencing (RNA-seq; data not shown) and translated to the amino acid sequence. Alignment was performed by the ClustalW program using the BioEdit Sequence Alignment Editor (http://www.mbio.ncsu.edu/BioEdit/bioedit.html). Identical resudes are shaded in gray. Putative RNA polymerase II C-terminal domain-interacting domain (CID), Nab3 binding domain, arginine-glutamate/arginine-serine (RE/RS) rich domain, RNA recognition motif (RRM), and Proline/Glutamine (P/Q) rich low-complexity domain (LCD) of ScNrd1 are indicated by colored lines. The RE/RS dipeptides of SpSeb1 and AkNrdA were shown in bold red font because they were outside of the RE/RS rich domain of ScNrd1.

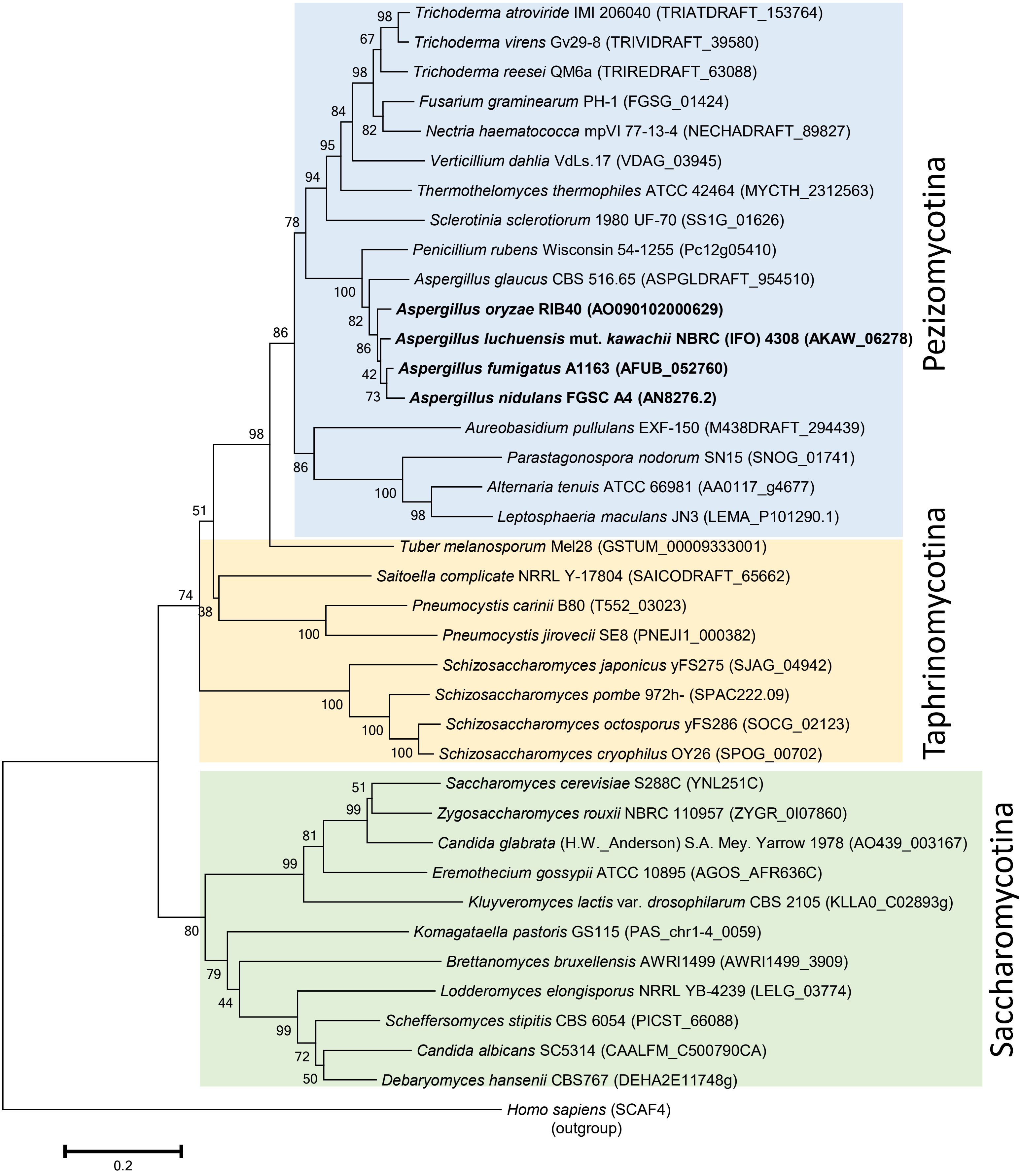

Supplementary Figure S2. Phylogenetic tree of amino acid sequences of *Saccharomyces cerevisiae* Nrd1-orthologs identified in subdivision Pezizomycotina, Taphrinomycotina, and Saccharomycotina.
The tree was constructed via the neighbor-joining method with complete gap deletion on MEGA version 6.0 (1). Bootstrap values (1,000 replicates) are indicated at the branches. The locus tags were shown in parenthesis. Bold-face indicate Nrd1-orhtologs characterized in this study.

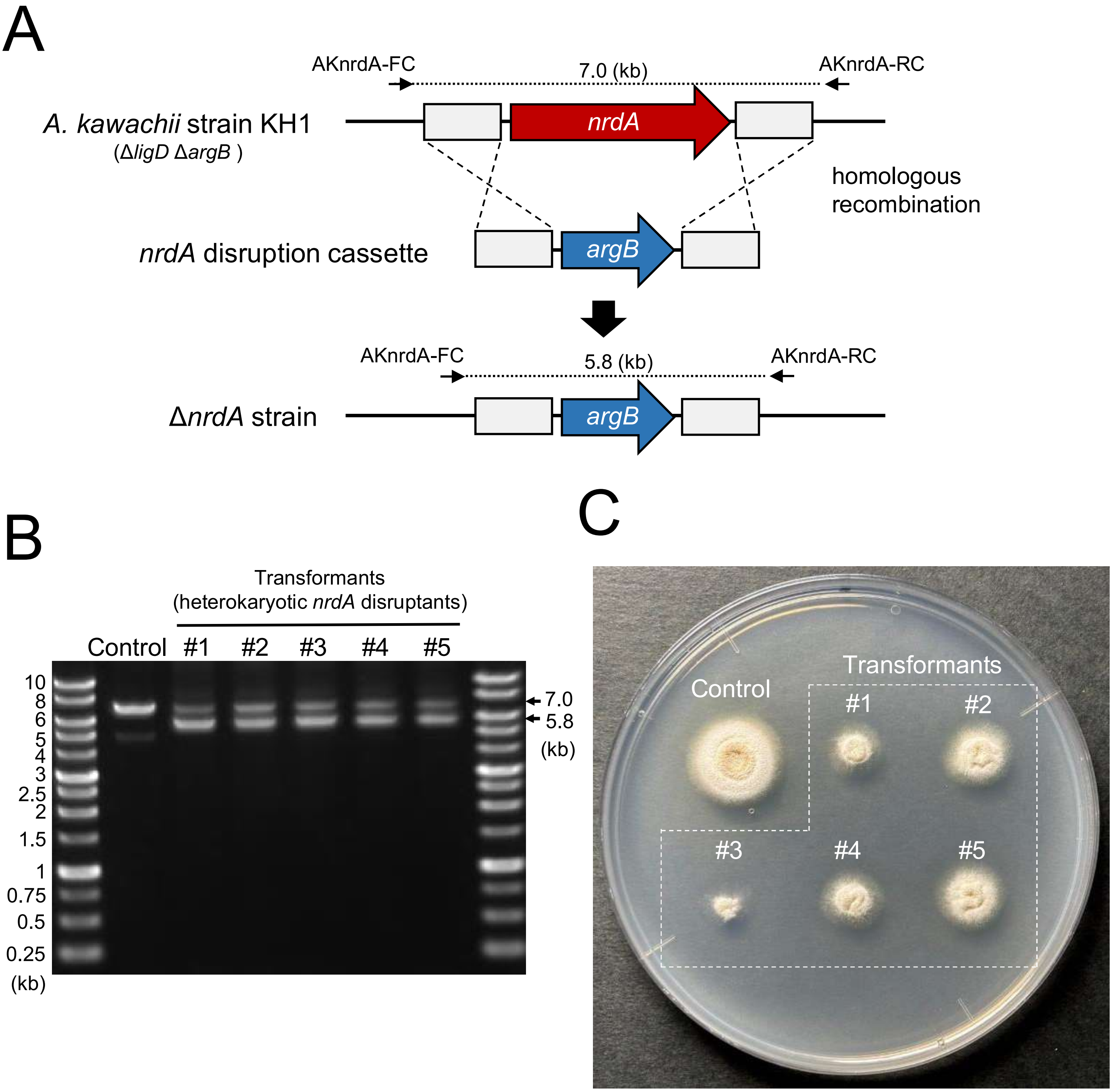

Supplementary Figure S3. Construction of *A. kawachii nrdA* disruptant strains.
Schematic representation of the introduction of *argB* marker in the locus of *nrdA* (A). A gene replacement cassette encompassing a homology arm at the 5' end of *nrdA*, *argB* marker and homology arm at the 3' end of *nrdA* locus was constructed through recombinant PCR using the primer pairs AKnrdA-FC/AKnrdA-R1, AKnrdA-F2/AKnrdA-R2, and AKnrdA-F3/AKnrdA-RC, respectively (Table S1). The resultant DNA fragment amplified with primers AKnrdA-F1/AKnrdA-R3 was used to transform the *A. kawachii* Δ*ligD* Δ*argB*, yielding the *A. kawachii* heterokaryotic *nrdA* disruptants. Agarose gel electrophoresis of PCR amplicons to confirm the introduction of the DNA cassettes in the transformants #1 to #5 (B). Colony morphology of the control and the transformants (heterokaryotic *nrdA* disruptants) (C). Conidia (1×10^5) were inoculated onto minimal agar medium and incubated at 30° C for 3 days. The result showed that the growth of heterokaryotic *nrdA* disruptants was reduced compared to the control strain. Microscopic observations indicated that most of the conidia from heterokaryotic *nrdA* disruptants failed to germinate in minimal liquid medium (data not shown). This is provably because the conidia of *A. kawachii* are mononuclear.

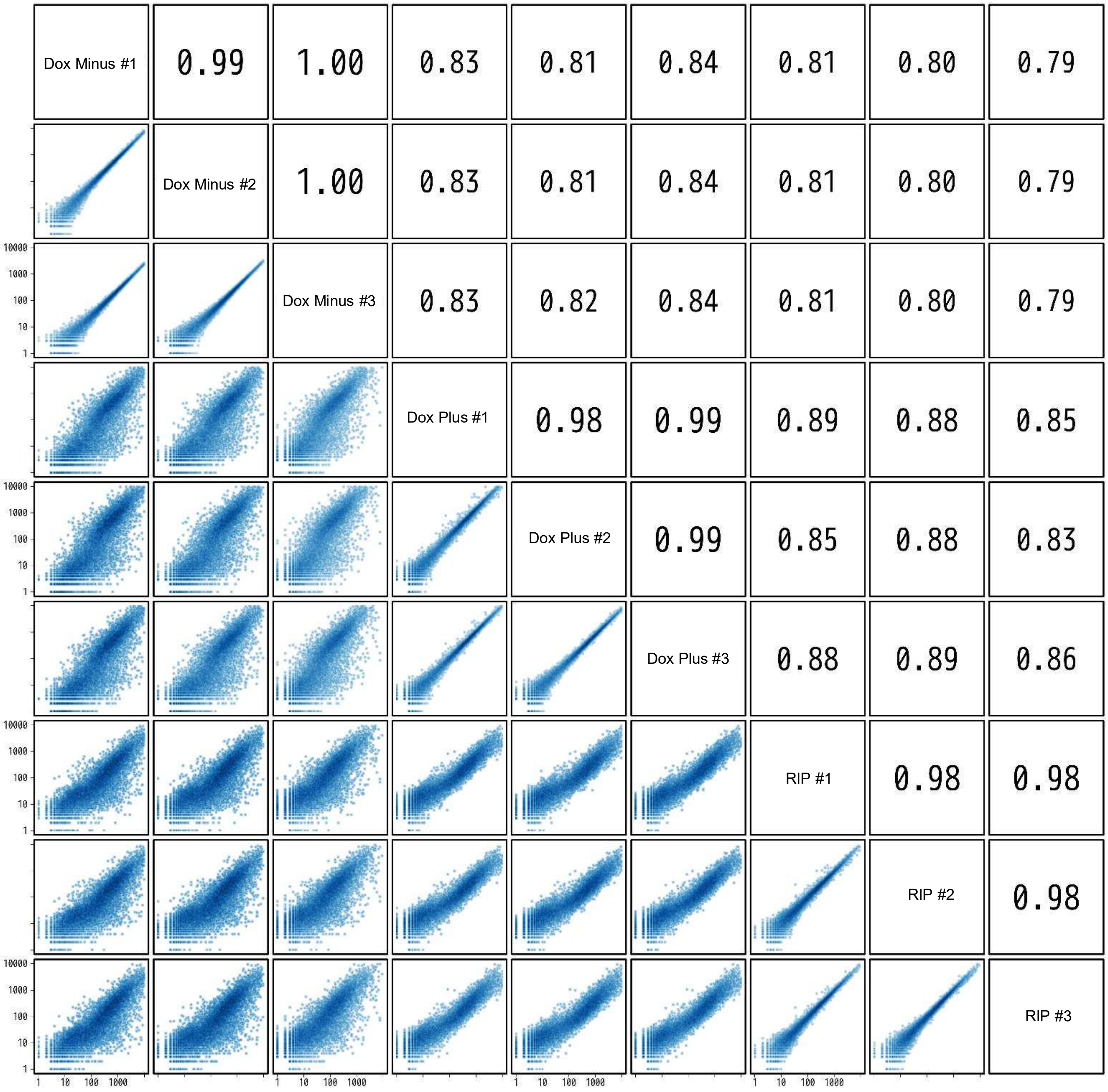

Supplementary Figure S4. Scatter plot and Pearson correlation coefficient for RIP-seq and RNA-seq data.

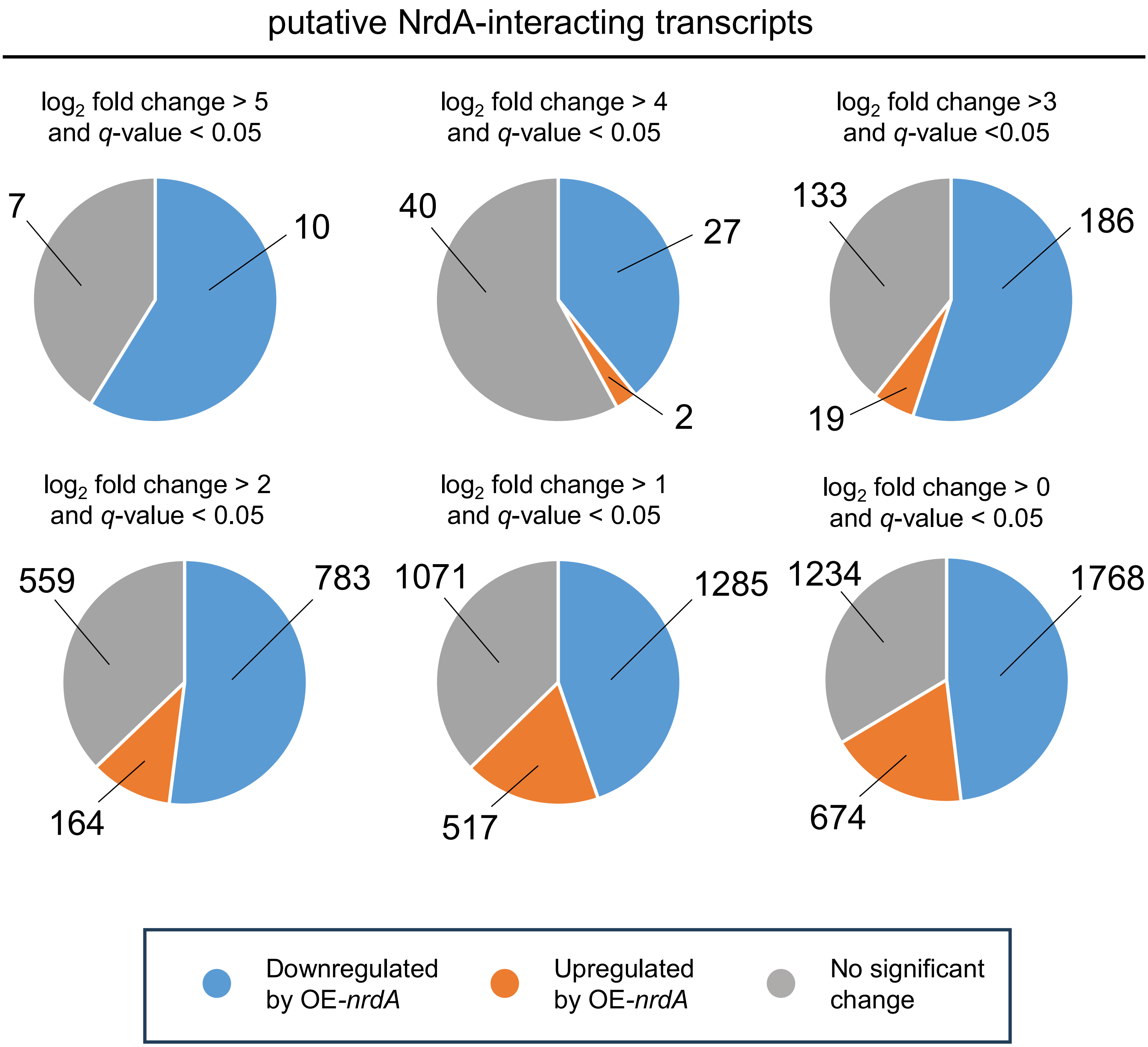

Supplementary Figure S5. Gene expression profiles of putative NrdA-interacting and transcripts due to *nrdA* overexpression.

The numbers of predicted NrdA-interacting transcripts are indicated by pie charts showing different levels of enrichment ($\log_2$ fold changes > 0, 1, 2, 3, 4, or 5; q-value < 0.05) determined by RNA immunoprecipitation. The upregulation, downregulation, or lack of significant changes due to the overexpression of S-NrdA were identified by RNA-seq analysis (q-value < 0.05).

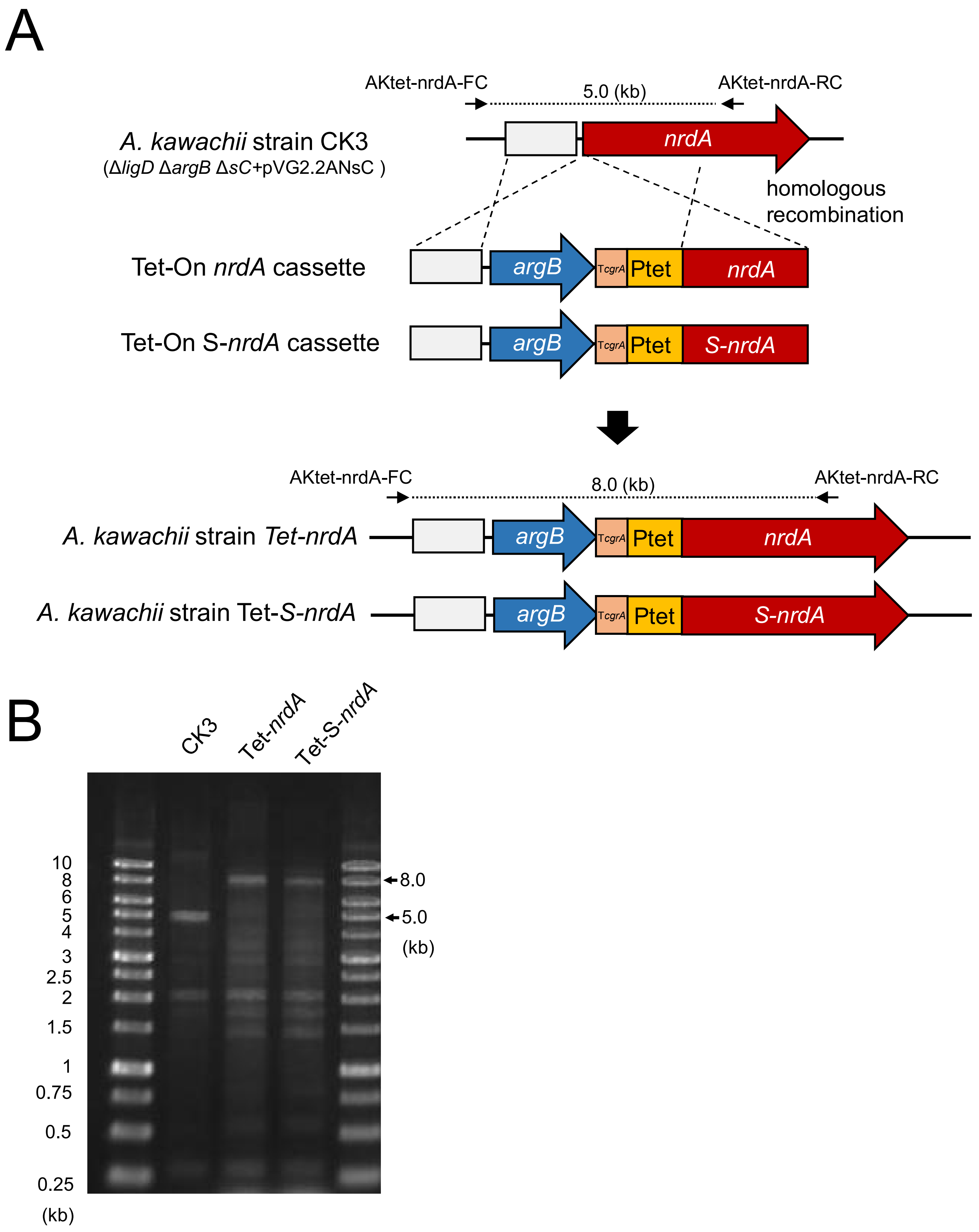

Supplementary Figure S6. Construction of *A. kawachii nrdA* conditional knockdown strains.
Schematic representation of the introduction of Tet-On promoter and N-terminal S-tagged *nrdA* cassettes in *nrdA* locus (A). Agarose gel electrophoresis of PCR amplicons to confirm the introduction of the DNA cassettes (B).

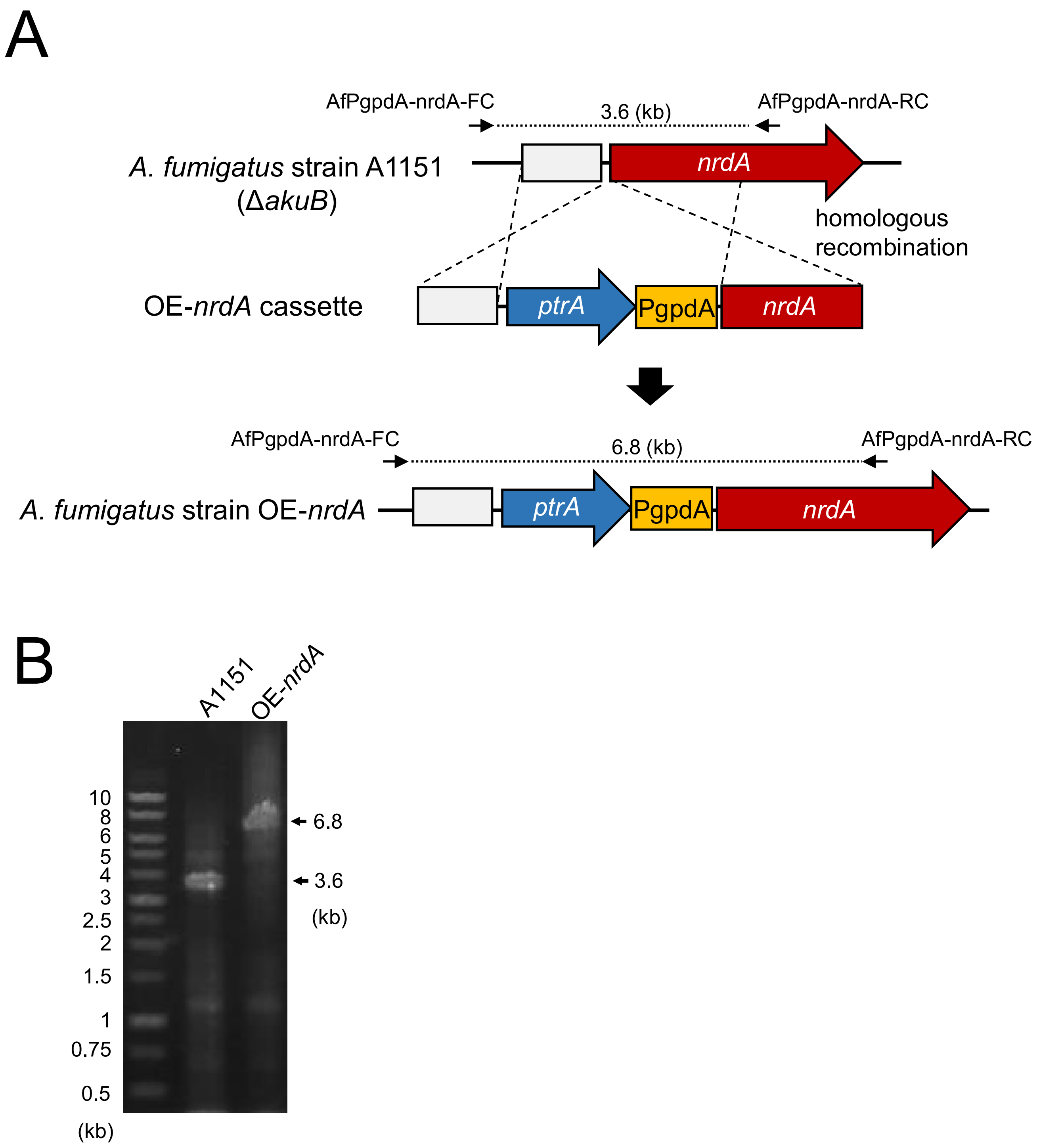

Supplementary Figure S7. Construction of *A. fumigatus nrdA* overexpression strain.
Schematic representation of the introduction of *gpdA* promoter in the upstream locus of *nrdA* (A). Agarose gel electrophoresis of PCR amplicons to confirm the introduction of the DNA cassettes (B).

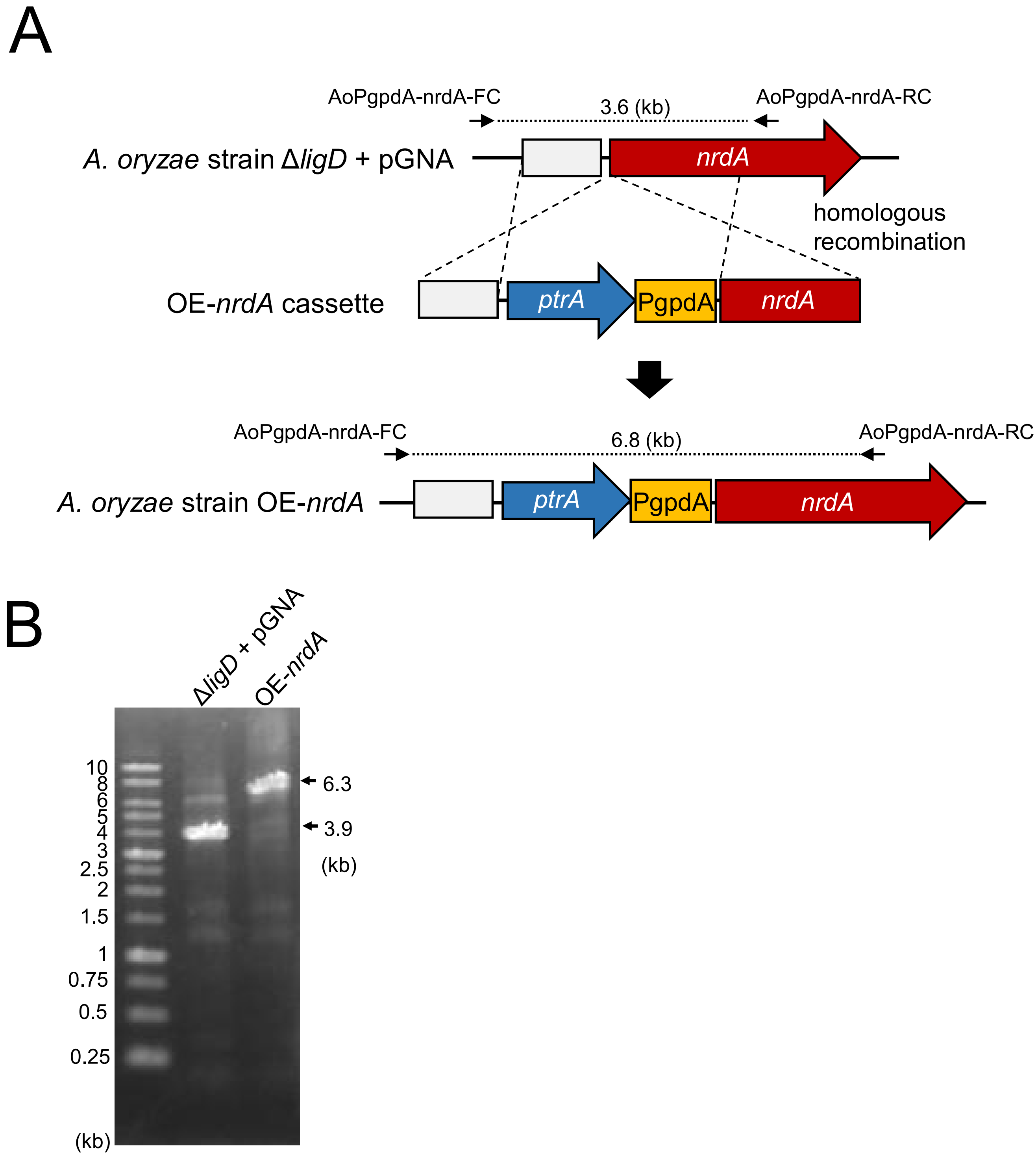

Supplementary Figure S8. Construction of *A. oryzae nrdA* overexpression strain.
Schematic representation of the introduction of *gpdA* promoter in the upstream locus of *nrdA* (A). Agarose gel electrophoresis of PCR amplicons to confirm the introduction of the DNA cassettes (B).

Table S1. PCR primers used in this study.

Primer name	Sequences (5'-3')	References
AKnrdA-FC (AKtet-nrdA-FC)	ATGGAGGAGGTACGGAGAGC	This study
AKnrdA-F1 (AKtet-nrdA-F1)	TCAAGGTGGAAGCCATGGCG	This study
AKnrdA-R1	GCATGCAAGCTTTCGCGAGCGGTGGACGAGATTTTTGGTTGTTG	This study
AKnrdA-F2	CAACAACCAAAAATCTCGTCCACCGCTCGCGAAAGCTTGCATGC	This study
AKnrdA-R2	GTCGTTCAAATAGCATGCTTCATAGAATTCGAGCTCGGTACCCGG	This study
AKnrdA-F3	CCGGGTACCGAGCTCGAATTCTATGAAGCATGCTATTTGAACGAC	This study
AKnrdA-R3	CTTCCGAAGGCTACCATGGC	This study
AKnrdA-RC	ACCACTGTTGCTGGTGTAGTTG	This study
AKtet-NrdA-R1	CTGCGTCCGGGTCTGTTTCAGGTGGACGAGATTTTTGGTTGTTG	This study
AKtet-NrdA-F2	CAACAACCAAAAATCTCGTCCACCTGAAACAGACCCGGACGCAG	This study
AKtet-NrdA-R2	GCGGAGAGAGATTCTTCTGCTGAAAGGCGCGAGTGAGATGCG	This study
AKtet-NrdA-F3	CGCATCTCACTCGCGCCTTTCAGCAGAAGAATCTCTCTCCGC	This study
AKtet-NrdA-R3	GTTCTGCTACAGCAGACGACATGGTGTTTAAACGGTGATGTCTGCTC	This study
AKtet-NrdA-F4	GAGCAGACATCACCGTTTAAACACCATGTCGTCTGCTGTAGCAGAAC	This study
AKtet-NrdA-R4	CTCACGACGTCTGTCCCAAC	This study
AKtet-NrdA-RC	GTACTCACCATAGACAGGGCTG	This study
AkS-nrdA-ptrA-R1	GCTGTCCATGTGCTGGCGTTCGAATTTAGCAGCAGCGGTTTCTTTGGCTCCAGCGCC TGCACCCATGGTGGACGAGATTTTTGGTTG	This study
AkS-nrdA-ptrA-F2	AACGCCAGCACATGGACAGCTCGTCTGCTGTAGCAGAACTG	This study
AkS-nrdA-ptrA-R2	ATGCAAGAGCGGCTCATCGTCTAATTGCTTCCTTGGCCTGG	This study
AkS-nrdA-ptrA-F3	CCAGGCCAAGGAAGCAATTAGACGATGAGCCGCTCTTGCAT	This study
AKtet-S-nrdA-R3	CCAGCGCCTGCACCCATGGTGTTTAAACGGTGATGTCTGCTC	This study
AKtet-S-nrdA-F4	GAGCAGACATCACCGTTTAAACACCATGGGTGCAGGCGCTGG	This study
pPTRI-gfp-nrdA-F1	CGTGATTGCGGTTGGAAAGTG	This study
pPTRI-gfp-nrdA-R1	GCGCCTGCACCAGCTCCCATGGTGGACGAGATTTTTGGTTG	This study
pPTRI-gfp-nrdA-F2	CAACCAAAAATCTCGTCCACCATGGGAGCTGGTGCAGGCGC	This study
pPTRI-gfp-nrdA-R2	CAGTTCTGCTACAGCAGACGATTTGTATAGTTCATCCATGCCATG	This study
pPTRI-gfp-nrdA-F3	CATGGCATGGATGAACTATACAAATCGTCTGCTGTAGCAGAACTG	This study
pPTRI-gfp-nrdA-R3	CTAATTGCTTCCTTGGCCTGG	This study
pPTRI-gfp-nrdA-inf-F	CCAAGCTTGCATGCCCGTGATTGCGGTTGGAAAGTG	This study
pPTRI-gfp-nrdA-inf-R	CCTCTAGAGTCGACCCTAATTGCTTCCTTGGCCTGG	This study
pPTRI-PgpdA-nrdA-inf-F1	CTCTAGAGGATCCCCACGGAGAAGCCCACCTTCAG	This study
pPTRI-PgpdA-nrdA-inf-R1	TCTGCTACTGCGGAAGACATTGTGATGTCTGCTCAAGCGG	This study
pPTRI-PgpdA-nrdA-inf-F2	CCGCTTGAGCAGACATCACAATGTCTTCCGCAGTAGCAGA	This study
pPTRI-PgpdA-nrdA-inf-R2	TCGAGCTCGGTACCCTCACTGACCAGGAGGTGGAG	This study
AfPgpdA-nrdA-F1	GTCACGAACCTTGGCCTTGC	This study
AfPgpdA-nrdA-R1	ATGCAAGAGCGGCTCATCGTGGCTGATGATTGTCGTTCAGG	This study
AfPgpdA-nrdA-F2	CCTGAACGACAATCATCAGCCACGATGAGCCGCTCTTGCAT	This study
AfPgpdA-nrdA-R3	CAACTCTGCTACAGCAGAAGACATTGTGATGTCTGCTCAAGCGG	This study
AfPgpdA-nrdA-F4	CCGCTTGAGCAGACATCACAATGTCTTCTGCTGTAGCAGAGTTG	This study
AfPgpdA-nrdA-R4	GTGAATCTGGACGGCTACCC	This study
AfPgpdA-nrdA-RC	CGCATGTAGTCGCGATCTCG	This study
AoPgpdA-nrdA-FC	GGAGATTGTCGGCGAATGTC	This study

Table S1 (continued). PCR primers used in this study.

Primer name	Sequences (5'-3')	References
AoPgpdA-nrdA-FC	GGAGATTGTCGGCGAATGTC	This study
AoPgpdA-nrdA-F1	CAGCGCAAACCATTGAAGGC	This study
AoPgpdA-nrdA-R1	ATGCAAGAGCGGCTCATCGTGGTTCCAGTACGGAGATATGGTTG	This study
AoPgpdA-nrdA-F2	CAACCATATCTCCGTACTGGAACCACGATGAGCCGCTCTTGCAT	This study
AoPgpdA-nrdA-R2	CTGAAGGTGGGCTTCTCCGTCGGGATCCCATTGGTAACGA	This study
AoPgpdA-nrdA-F3	TCGTTACCAATGGGATCCCGACGGAGAAGCCCACCTTCAG	This study
AoPgpdA-nrdA-R3	GTTCTGCTACAGCAGACGACATTGTGATGTCTGCTCAAGCGG	This study
AoPgpdA-nrdA-F4	CCGCTTGAGCAGACATCACAATGTCGTCTGCTGTAGCAGAAC	This study
AoPgpdA-nrdA-R4	GAGTCGCCATGGTACTCACC	This study
AoPgpdA-nrdA-RC	GTGGCGGTAGTGTAGGGTCC	This study
ANnrdA-RT-F	TCCTCTGAGCGCATTAAGCC	This study
ANnrdA-RT-R	GCCGCTAAGGGATTCTGAGG	This study
ANipnA-RT-F	TCCCTACCCCGAGGCTGCTATCAAGACG	2
ANipnA-RT-R	CATTTCACCCGATGGATGGGCGCTTT	2
ANaflR-RT-F	ATGGAGCCCCCAGCGATCAGCCAG	2
ANaflR-RT-R	TTGGTGATGGTGCTGTCTTTGGCTGCTCAAC	2
ANstcU-RT-F	GTCCTCCTCCGATAATTACCGTC	This study
ANstcU-RT-R	GGTGACGGCATCAGGGTCAC	This study
ANacnA-RT-F	GACGGATACGGCAACACCAAG	This study
ANacnA-RT-R	GCTCTTGGTGTGAATGACGGTG	This study
AFnrdA-RT-F	CACGCAGCAAGATCGATTCC	This study
AFnrdA-RT-R	GGGGTACGCCGACGTTATTG	This study
AFfumR-RT-F	GACGTCAATCGTAAGCGAATGG	This study
AFfumR-RT-R	CTGACCGGCGCAGAATGTATG	This study
AFhelA-RT-F	CTGGCTCGGTCTACCGTATG	This study
AFhelA-RT-R	CAGTGTCCATCGGCTGTCTG	This study
AFpyr2-RT-F	GCTTCTTCCCCTGCGAAACTC	This study
AFpyr2-RT-R	CTGGACGTTTGTCGTGCCATG	This study
AFact1-RT-F	TCACTGCCCTTGCTCCCTCGTC	This study
AFact1-RT-R	GCACTTGCGGTGAACGATCGAA	This study
AOnrdA-RT-F	GAGTCCACCACACCTGAGGG	This study
AOnrdA-RT-R	GCCATATCAGCCAGGGCTTTC	This study
AOkojR-RT-F	CGGTATATGCGGTCCTGGATC	This study
AOkojR-RT-R	GTGGCCTGAGTACAAGGAGC	This study
AOkojA-RT-F	GGCCTCATCAGCGCATACAATC	This study
AOkojA-RT-R	GGTGTGGATGATACGGTCGAC	This study
AOkojT-RT-F	TCATTGTCGGGTTCGATGGC	This study
AOkojT-RT-R	AGTGGAGTCGATGGAGGAAGC	This study
AOipnA-RT-F	CCACTTTAAGAAGGATGACGCCC	This study
AOipnA-RT-R	GAGCGACACATCTTCATGCC	This study
AOactA-RT-F	GCTCCGTGTTGCTCCCGAG	3
AOactA-RT-R	GCCTGGATGGAGACGTAGAAGG	3

Table S2. The highly enriched transcripts by RIP of S-NrdA-mRNA complex (surrounded by a dotted line in Fig. 4B).

Locus tags	Annotation in DDBJ/ENA/GenBank database	Results of significant Pfam-A matches [a]	homologs in *S. cerevisiae* S288C	homologs in *Schiz. pombe* 972h-	RIP-seq of Dox plus condition/ RNA-seq of DOX plus condition	RNA-seq of DOX plus condition/ RNA-seq of DOX minus condition
			BLASTP top hit [b] (Identities, e-values)	BLASTP top hit [b] (Identities, e-values)		
AKAW_10564	Metallo-beta-lactamase domain protein	Lactamase_B (Metallo-beta-lactamase superfamily)	no hit	no hit	5.88	-1.77
AKAW_10304	Hypothetical protein	Git3 (G protein-coupled glucose receptor regulating Gpa2)	no hit	no hit	5.71	-1.95
AKAW_06494	Hypothetical protein	n.d.	no hit	no hit	5.61	-2.41
AKAW_10693	Hypothetical protein	n.d.	no hit	no hit	5.55	-4.77
AKAW_01947	Hypothetical oxidoreductase	ADH_zinc_N (Zinc-binding dehydrogenase); ADH_N (Alcohol dehydrogenase GroES-like domain)	YNL134C (31% [117/381], 7e-43)	SPAC2E1P3.01 (29% [105/363], 4e-39)	5.37	-1.39
AKAW_10686	Tannase	Tannase (Tannase and feruloyl esterase)	no hit	no hit	5.25	-2.35
AKAW_10741	Hypothetical protein	n.d.	no hit	no hit	5.22	-3.59
AKAW_04699	Hypothetical protein	Tannase (Tannase and feruloyl esterase)	no hit	no hit	5.12	-2.19
AKAW_10588	Hypothetical protein	n.d.	no hit	no hit	5.10	-5.09
AKAW_02137	Hypothetical protein	n.d.	no hit	no hit	5.01	-2.09

[a] Domain search was performed by Pfam (http://pfam.xfam.org/).

[b] BLASTP analysis was performed using NCBI BASTP (https://blast.ncbi.nlm.nih.gov/Blast.cgi).

References

1. Tamura K, Stecher G, Peterson D, Filipski A, Kumar S. 2013. MEGA6: Molecular Evolutionary Genetics Analysis version 6.0. *Mol Biol Evol* **30**:2725–2729.

2. Atoui A, Bao D, Kaur N, Grayburn WS, Calvo AM. 2008. *Aspergillus nidulans* natural product biosynthesis is regulated by MpkB, a putative pheromone response mitogen-activated protein kinase. *Appl Environ Microbiol* **74**:3596–600.

3. Nakamura E, Kadooka C, Okutsu K, Yoshizaki Y, Takamine K, Goto M, Tamaki H, Futagami T. 2021. Citrate exporter enhances both extracellular and intracellular citric acid accumulation in the koji fungi *Aspergillus luchuensis* mut. *kawachii* and *Aspergillus oryzae*. *J Biosci Bioeng* **131**:68–76.
